# Spontaneous object recognition: a promising approach to the comparative study of memory

**DOI:** 10.3389/fnbeh.2015.00183

**Published:** 2015-07-10

**Authors:** Rachel Blaser, Charles Heyser

**Affiliations:** ^1^Department of Psychological Sciences, University of San DiegoSan Diego, CA, USA; ^2^Behavioral Testing Core, Department of Neurosciences, University of California, San DiegoSan Diego, CA, USA

**Keywords:** object, exploration, recognition, memory, novelty, neophobia

## Abstract

Spontaneous recognition of a novel object is a popular measure of exploratory behavior, perception and recognition memory in rodent models. Because of its relative simplicity and speed of testing, the variety of stimuli that can be used, and its ecological validity across species, it is also an attractive task for comparative research. To date, variants of this test have been used with vertebrate and invertebrate species, but the methods have seldom been sufficiently standardized to allow cross-species comparison. Here, we review the methods necessary for the study of novel object recognition in mammalian and non-mammalian models, as well as the results of these experiments. Critical to the use of this test is an understanding of the organism’s initial response to a novel object, the modulation of exploration by context, and species differences in object perception and exploratory behaviors. We argue that with appropriate consideration of species differences in perception, object affordances, and natural exploratory behaviors, the spontaneous object recognition test can be a valid and versatile tool for translational research with non-mammalian models.

The spontaneous object recognition test is widely used as a measure of memory in rodents (Bevins and Besheer, 2006; Dere et al., 2007; Ennaceur, 2010). In a prototypical design, the animal is first exposed to a sample object (or two identical samples), and subsequently tested with an object identical to the sample (familiar object) and a novel one. In rodents, the natural tendency to explore novelty leads to a preference in the test for the novel over the familiar test object, which demonstrates recognition memory of the familiar object (Bevins and Besheer, 2006). The task is appealing for researchers in behavioral neuroscience because it does not require food or water deprivation, it is not aversively motivated, and the duration of training and testing is relatively short. This may help reduce confounds when administering treatments that could affect sensory, motor, or motivational responses to a reinforcer in a conditioning task, and it may also more closely resemble the conditions under which human cognition is typically studied (Ennaceur, 2010). One difficulty in comparing results of reinforced learning and memory tasks across widely divergent species is the problem of equating motivation in the experimental setting, including hunger, response to stress, or pain sensitivity. The spontaneous object recognition test may be appealing for comparative research due to the simplicity of the task, ecological relevance to a wide variety of species, and limited need for motivational manipulations.

The use of non-mammalian animal models to study cognition is nothing new—birds were among the first animal models of learning (Thorndike, 1911), and many classic experiments on memory and visual perception used pigeons and chicks (e.g., Guttman and Kalish, 1956; Jenkins and Harrison, 1962; Honig et al., 1963; Herrnstein and Loveland, 1964; Roberts, 1972; Lubow, 1974). More recently, according to Shettleworth (2009), roughly 1/3 of experiments published in the Journal of Comparative Psychology (JCP) from 2005–2007 used nonmammalian species. Currently, our understanding of pigeon visual cognition may surpass that of any other non-primate species, certainly that of rodents (Soto and Wasserman, 2014). The contribution of pigeon research to the field of visual cognition should yield little doubt about the utility of non-mammalian models of human cognition. However, because birds are less amenable to common genetic and neuroscientific procedures than rodents, their utility has been largely underappreciated in the field of neuroscience. Despite the challenges of working with non-standard animal models in a laboratory setting, birds and other species including fish, amphibians, reptiles, and invertebrates each have advantages for comparative research and may merit further attention (see Table 1). We propose that the exploration and recognition of novel objects may provide an interesting and useful direction for comparative research with non-mammalian models.

**Table 1:** **Advantages of some exemplary non-mammalian species as model organisms**.

	Birds				Reptiles		Fish		Invertebrates					
	Chicken	Crow	Pigeon	Zebra finch	Anole	Painted turtle	Zebrafish	Stickleback	Sea hare	Cockroach	Octopus	Jumping Spider	Fruit fly	Honeybee
**Technical**
Low cost	x				x		x	x	x	x		x	x	x
Ease of husbandry	x		x	x			x	x	x				x	x
**Neurobiology**
Genome sequenced	x		x	x	x	x	x	x	x				x	x
Genetic variants available							x						x
Well-characterized nervous system	x		x	x			x	x	x	x			x	x
Brain/Neuron size	x	x	x						x		x
Well-characterized development	x			x			x						x
**Behavior**
Well-characterized sensory system	x		x	x			x	x			x		x	x
Highly-developed visual system	x	x	x	x			x				x	x		x
Sophisticated motor repertoire	x	x	x	x	x	x	x	x		x	x	x		x
Established response to novelty		x					x	x		x	x

There are two common approaches to the comparative study of cognition. Typically in the neurosciences, two distantly related species sharing a behavioral trait of interest are compared. This is usually done with the expectation that the neurological mechanisms are homologous to some degree but simplified in one of the species under investigation. For example, the study of spatial learning in rats, fear/anxiety in zebrafish, and habituation in *Aplysia*, is often done with the goal of identifying mechanisms that are directly translatable to humans (whether at the genetic, molecular, cellular, or systems level). In these cases, the comparative model is typically chosen based on methodological advantages, such as the rapid reproductive cycle of drosophila (for genetic analysis), the transparency of developing zebrafish embryos (for developmental analysis), or the simplified and well-mapped nervous system of *Aplysia* (for synaptic-level analysis). However, this approach can also be quite productive even when the degree of homology is unknown, or when convergent evolution is assumed. The pigeon visual system is neuroanatomically quite different from the primate visual system, but pigeon research has nonetheless been quite valuable to our understanding of object perception and recognition.

An alternative approach is to compare two closely related species that differ specifically in a behavioral characteristic of interest. This approach is analogous to the invasive methods of lesioning or genetic knock-outs, in that two highly similar systems are compared which differ in the feature of interest. The study of hormones involved in pair-bonding and parenting in voles is one example of this approach applied successfully to neuroscience (e.g., Young and Wang, 2004). Another example is the study of spatial cognition in food-caching and non-caching birds, which differ both in cognitive (e.g., spatial memory) and neurophysiological (e.g., hippocampal volume) domains (e.g., Basil et al., 1996). In these cases, the comparative model is typically selected on the basis of a highly specialized trait, such as song-learning in zebra finches, food-caching in corvids, or pair-bonding in voles. The primary advantage of the comparative method is that the behavior of healthy, intact animals can be observed in all conditions. A major disadvantage is that the method is correlational, but it still makes a useful complement to other experimental techniques.

## Background

The study of object exploration is partially derived from two historical lines of research. The first is the study of curiosity in animals (Darwin, 1874; Kinnaman, 1902; Thorpe, 1956; Berlyne, 1960; Berlyne et al., 1966; Barnett and Cowan, 1976); the second is the broader consideration of investigatory behavior and its practical implications. With the exceptions of Small (1899), Slonaker (1912) and Hall and Ballachey (1932) there were very few studies concerning the investigatory behavior of the laboratory rat in the first half of the 20th century (Barnett and Cowan, 1976). Barnett and Cowan (1976) noted in their review of exploration that, “…apparently undirected wandering (of animals) were disregarded as an important object of study.” In fact, exploratory behavior during laboratory experiments has often been viewed as a nuisance phenomenon that may interfere with the behavior of interest. For instance, Chance and Mead (1955) observed that a hungry rat spent significant time investigating a novel feature of the environment before eating. Cohen and Stettner (1968) reported that after learning to navigate a straight runway, water-deprived rats explored a newly introduced blind alley before they finally approached and consumed the reinforcer. Because of this, researchers often take steps to eliminate exploratory behavior by familiarizing the animal to the apparatus and experimenters.

Early information regarding object exploration came from an unusual source: studies aimed at population control (i.e., how to build a better mouse trap…). For example, it was reported that Norway rats usually travel in established pathways to avoid unfamiliar objects (Minckler and Peaseh, 1938; Orgain and Schein, 1953). This may explain, in part, the practical and methodological problems in trapping rats in a stable environment (Chitty, 1954), as wild Norway rats probably avoid traps just as they avoid most new objects (Barnett, 1958). In contrast, studies investigating wild rats on rubbish tips, where the refuse is frequently moved and buried, show little reaction to traps; rats are caught within 2 h (Boice and Boice, 1968). This neophobia was surprising since it had been well documented that laboratory strains of rats are noted for their “curiosity” and “inquisitiveness” (Berlyne, 1960). Barnett (1958) reported the contrast between the neophobic behavior of caged wild rats and the neophilic response of laboratory strains. The behavior of wild rats was termed the “new-object reaction,” which involves the avoidance of unfamiliar objects in familiar surrounding (Shorten, 1954). Cowan (1976) reported that rats did not avoid objects in a residential environment if the objects were present when the rats were first introduced; but such objects evoked “new object reactions” avoidance if introduced after the rats had adapted to their new environment. New-object reactions have been reported in wild mice (Southern, 1954; Wolfe, 1969) and in Voles (Shillito, 1963). However, unlike new objects, new areas are quickly explored by both wild and laboratory rats (Barnett, 1975). When given the choice between novel and familiar environments, subjects often choose to spend more time in the novel environment (Montgomery, 1954; Dember, 1956; Fowler, 1958; Hughes, 1965, 1968). The significance of these studies is to highlight differences between wild and laboratory animals and further to illustrate the dependency of the animal’s reaction on the prevailing environmental conditions (familiar vs. unfamiliar and changing vs. unchanging).

Recognition of novel objects has since become a widely used measure of learning and memory in rodents (Berlyne, 1960; Ennaceur and Delacour, 1988; Ennaceur, 2010). The standard object recognition test relies on the behavioral tendency of rodents to seek out and explore novelty (Berlyne et al., 1966; Barnett and Cowan, 1976; Ennaceur and Delacour, 1988; Bardo et al., 1993). Novel stimuli, such as unfamiliar environments or objects, can evoke both approach and avoidance behaviors (Montgomery, 1955; Dember, 1956; Welker, 1959; Berlyne, 1960), but there is evidence that the motivational strength of novelty can be quite high. For example, Nissen (1930) reported that rats would cross an electrified grid to gain access to a complex maze (containing novel objects) that they could enter and explore. One of the initial papers by Berlyne (1950) provided a methodology for the study of recognition memory in female Long-Evans rats using objects. Using a variety of procedures, similar results have been found in gerbils (Cheal, 1978), marmosets (Menzel and Menzel, 1979), and baboons, (Joubert and Vauclair, 1986). Changes in spatial orientation (by re-arranging the objects) have also been used to study recognition memory in hamsters (Poucet et al., 1986) and in Mongolian gerbils (Wilz and Bolton, 1971). With the publication by Ennaceur and Delacour (1988), the novel object recognition procedure has become a popular method for studying memory because there is no explicit need for food or water restriction, and several behavioral endpoints can be rapidly obtained, including general activity, reactivity to novelty, and learning. The novel object recognition test has been studied in many different mammalian species including (but not limited to) mice (Şık et al., 2003; Kim et al., 2005), rats (Berlyne, 1950; Ennaceur and Delacour, 1988), hamsters (Thinus-Blanc et al., 1992), dogs (Callahan et al., 2000), monkeys (Zola-Morgan et al., 1983) and Gottinger minipigs (Kornum et al., 2007).

The standard procedure is used to measure recognition memory on a relatively short time scale (usually minutes-hours). In the prototypical design, the animal is familiarized to an object, which is then replaced by a novel object in the test (Berlyne, 1950; Ennaceur and Delacour, 1988). Assuming that confounding biases and object preferences are ruled out (see “Design Considerations” Section), any discrimination in a test between a novel and a familiar object by definition indicates some form of memory (Bevins and Besheer, 2006). However, several variants of this task have been used to target more specific cognitive processes. For example, one popular version of this task is used to assess spatial memory by re-arranging familiar objects into a different geometric pattern (Aggleton et al., 1986; Poucet et al., 1986; Dix and Aggleton, 1999). The memory mechanisms appear to differ between the variants of this task, as a dissociation has been reported in the underlying neurobiology with recognition memory for individual items relying on the perirhinal cortex and memory for spatial variants of this test involving the hippocampus (Aggleton et al., 1986; Ennaceur et al., 1996; Aggleton et al., 1997; Bussey et al., 1999; Barker and Warburton, 2009). However, there is still much to be learned about the cognitive mechanisms by which discrimination occurs in the variants of this task (Bevins and Besheer, 2006; Ennaceur, 2010). Recently variations in the procedure have been reported to measure a diversity of constructs including neophobia (Powell et al., 2004), boldness (Toms and Echevarria, 2014), novelty-seeking (Powell et al., 2004), attention (Braida et al., 2014), spatial memory (Poucet et al., 1986; Thinus-Blanc et al., 1992), working memory (Aggleton et al., 1986), and episodic memory (Eacott and Norman, 2004; Dere et al., 2005, 2007; Kesner and Hunsaker, 2010). Thoughtful experimental design and thoroughly validated procedures are necessary in order to use this test for the study of anything more specific than “recognition memory.” For example, in order to establish that dishabituation in response to the change in location of an object truly measures spatial memory, evidence must be provided that the animal perceives the objects as invariants against different visual backgrounds and from different approach trajectories. Similarly, in order to establish that a subject remembers the context in which an object has been encountered, simple perceptual interactions between the object and its background must be ruled out (e.g., Eacott et al., 2005).

## Non-Mammalian Models

Despite its popularity with rodent models, the novel object recognition test has only been used sporadically with non-mammalian subjects (Miletto Petrazzini et al., 2012; Braida et al., 2014; Lucon-Xiccato and Dadda, 2014). Individual components of the test have been examined independently, however, providing a useful foundation for future research. In particular, visual object perception in pigeons has been studied in depth (Soto and Wasserman, 2014), as has the development and neurophysiology of the zebrafish visual system (Bilotta and Saszik, 2001; Renninger et al., 2011; Chhetri et al., 2014). Initial behavioral responses toward a novel object have been studied in birds and fish as well (e.g., Mettke-Hofmann et al., 2002; Burns, 2008; Toms and Echevarria, 2014). While explicit measures of familiarization to an object and subsequent memory for that object are few, the thorough characterization of object perception and response to novelty will be useful for interpreting the results of learning and memory tests, and may even provide a stronger foundation for such research than currently is available with rodents, in whom the mechanisms of object perception are still rather poorly understood.

### Object Perception

Perhaps the most fundamental component of novel object recognition is the perceptual process by which three-dimensional objects are identified and discriminated from other stimuli in the environment. Inherent in this process is categorization, or the ability to respond similarly toward non-identical objects (e.g., two different food items) or non-identical views of the same object. The ability to categorize objects may be useful to produce appropriate behavioral responses to food, predators, and conspecifics, among other stimuli. One simple solution to the problem is to produce a fixed response to certain “sign stimuli” that are shared by members of the category. For example, a predator might strike indiscriminately at any moving object within certain parameters of size and speed (Ewert, 1987), or a rat may engage in antipredatory responses in the presence of an odor that signifies a predator (Blanchard et al., 2001). A relatively inflexible response to specific stimulus features could produce adaptive behavior that looks like object categorization if all members of the category (all prey items, all predators) present the relevant feature.

While this type of inflexible responding does occur throughout the animal kingdom, many species are able to recognize objects that appear quite different from different angles or are partially occluded, and to learn to categorize objects that have relatively few stimulus features in common. In humans, the visual system plays a central role in object recognition. Human object recognition has been reviewed extensively elsewhere (e.g., Biederman and Gerhardstein, 1993; Tarr and Bülthoff, 1995; Ungerleider and Bell, 2011; Wallis, 2013), but a few attributes of human object recognition are worth noting for comparative purposes. First, humans are able under many conditions to perceive objects as invariant, even when the object is rotated, partially occluded, or presented in different contexts such as background, illumination, or scale. Humans can correctly assign novel exemplars to a category (e.g., an unfamiliar dog elicits a “dog” response). Humans are also able to recognize that a two-dimensional image corresponds to a three-dimensional object, and simultaneously distinguish between them [e.g., recognize a strawberry in a photograph or drawing, but not attempt to eat the photograph (Spetch and Friedman, 2006a)].

Because the pigeon also has a highly developed visual system, it has been a popular animal model for the study of visual perception (Spetch and Friedman, 2006a; Soto and Wasserman, 2014; Castro et al., 2015). Pigeons do not show the same degree of object invariance as humans, although performance on such tasks improves with a larger array of training exemplars (Soto and Wasserman, 2014). Pigeons exhibit a systematic decrease in recognition of an object (i.e., a generalization decrement) as the test image is rotated further from the view(s) used for training (Spetch and Friedman, 2006a)—this is sometimes also true of humans, but to a lesser degree. Pigeons also appear to recognize a correspondence between actual 3D objects and pictures of those objects, with some degree of positive transfer in both directions, from 3D to pictures and* vice versa* (Spetch and Friedman, 2006b). Soto and Wasserman have argued that many aspects of object recognition in pigeons can be explained by an error-correction algorithm that could be shared with humans and other amniotic vertebrates (Soto et al., 2012).

Visual object perception has been examined in non-avian models as well, to a much lesser extent. Teleost fish and sharks can learn through operant conditioning to discriminate between complex two-dimensional images, and respond to novel exemplars and novel rotations of learned objects (Schluessel et al., 2012, 2014; Fuss et al., 2014; Schluessel and Duengen, 2015). Only very basic visual discrimination has been studied in zebrafish (e.g., Bilotta et al., 2005; Colwill et al., 2005), but research on the genetics and development of their sophisticated visual system is already of proven translational value to human visual disorders (Baier, 2000; Maurer et al., 2011; Gestri et al., 2012). The ongoing study of zebrafish neurobiology will provide a useful foundation for more complex learning and behavioral questions. Reptiles, amphibians, and a variety of invertebrates have also demonstrated the capacity to discriminate between complex visual stimuli, but their translational relevance to mammals is not yet established (e.g., Sutherland et al., 1963; Powers, 1990; Wilkinson et al., 2013).

Of course, pigeons were selected for the study of visual object recognition precisely because of their sophisticated visual system. Other species explore and identify objects using a variety of sensory systems including electrolocation, echolocation, and tactile, olfactory, and gravipositional perception. Given the tendency of human scientists to design experiments around visual stimuli, most likely we still underestimate the role that non-visual senses play in object recognition (Vasconcelos et al., 2011). Drosophila, for example, explore objects in their environment using not only their visual system but also gravipositional cues that promote exploration of (climbing on) taller, steep objects (Robie et al., 2010). Senegal parrots explore objects using not only vision but tactile organs in the bill tip (Demery et al., 2011). *Gnathonemus peterseii*, an electric fish, changes the emission frequency of its electric organ discharges when it encounters a novel object in its environment, and can discriminate stimulus features such as volume, substance (i.e., metal vs. plastic), and shape (e.g., rounded vs. square), using electrical signals (von der Emde, 1990, 1999, 2006; von der Emde and Fetz, 2007). Obviously, species differences in perception must be considered in order to select appropriate objects and to define and measure exploration in this test.

### Object Exploration

In order to determine whether an object has been detected and identified either as novel or familiar, a measurable behavioral response is required. Much of the classic object learning research uses reinforcement to encourage a measurable behavior (e.g., pecking at a choice button). The novel object recognition task is unusual in that it does not use any extraneous reinforcement to shape behavior. Therefore, a second major component of the novel object recognition test is the subject’s intrinsic response to novelty—typically exploration, avoidance, or a combination of the two (Hughes, 1997). Exploration of novel objects is modulated by attributes of the subject, attributes of the object, and the context in which the object is encountered.

#### Subject Attributes

Subject attributes that have been examined include the species, age, sex, development/experience, and individual predisposition (sometimes studied as personality, temperament, trait, or behavior syndrome). The most fundamental subject attribute is previous experience, which is central to the very definition of novelty. Berlyne (1960) distinguished absolute novelty, which involves some quality never previously experienced from relative novelty of familiar items arranged in an unfamiliar way. Relative novelty can be operationally defined only with reference to the past experience of the animal. Therefore, “novelty” is not a quality of stimuli *per se* and “cannot be distinguished (from other stimuli) by physicochemical properties” (Berlyne, 1960, p 20), but is an expression that refers to “an interaction between stimulus and perceiver” (Dember, 1960, p 348) with respect to an organism’s past experiences with the stimuli in question. “Unlike objectively measurable qualities such as brightness, shape, and texture of stimuli, novelty is defined in terms of the extent to which stimuli have been previously experienced and is therefore specific to individuals” (Hughes, 2007).

In birds, age has been repeatedly shown to affect the latency and duration of novel object exploration. In *Milvago chimango*, a neotropical raptor, juveniles (<1 year in age) approach a novel object more quickly and explore it for longer than adults (Biondi et al., 2010, 2013, 2015). Juvenile ravens, in contrast, explore objects more at 6 months of age than 3 months of age (Stöwe et al., 2006). In Gouldian finches as well, exploration time is negatively correlated with age (Mettke-Hofmann, 2012). Ecological differences between bird species appear to also affect object exploration. In a large-scale study comparing over 70 species of parrots, exploration time was shown to be species-specific (within-species differences in approach latencies were smaller than between-species differences), with 25% of the variance accounted for by habitat complexity (Mettke-Hofmann et al., 2002). Species differences in other birds have also been observed based on migratory patterns and habitat (Echeverría et al., 2006; Echeverría and Vassallo, 2008; Nilsson et al., 2010; Mettke-Hofmann et al., 2013).

In fish, most of the research on novel object exploration is driven by an interest in individual differences, and tested whether the tendency to approach a novel object is correlated with other behaviors such as aggression, antipredatory behaviors, and other exploratory behaviors (Burns, 2008; Conrad et al., 2011; Toms and Echevarria, 2014). The results with several species of fish, most frequently zebrafish and guppies (Wilson and Godin, 2009; Brown and Irving, 2013; Toms and Echevarria, 2014), have indicated that exploration of novel objects is generally a reliable behavior—that is, individuals’ exploration scores in two separate tests are significantly correlated, and often measures of exploration (i.e., latency to approach and duration of inspection) are correlated with each other (Jones and Godin, 2009; Toms and Echevarria, 2014). However, despite widespread testing, there is little indication that exploration of a novel object is systematically related to any other behaviors, even other exploratory behaviors such as exploration of a novel environment or predator inspection (Burns, 2008; Dahlbom et al., 2011; Toms and Echevarria, 2014). Correlations have frequently been reported, such as a relationship with shelter emergence in *Brachyrhaphis episcopi* and bluegill sunfish (Brown and Braithwaite, 2004; Wilson and Godin, 2009), time spent in the center of an open field (Dahlbom et al., 2011) and shelter emergence (Toms and Echevarria, 2014) in zebrafish, and predator approach and general activity in guppies (Smith and Blumstein, 2010). However, such correlations are often sporadic and do not appear consistently across experimental replications. While this may be due to the widely varying procedures for testing object exploration in fish, there is little reason at this point to believe that novel object exploration is part of a larger “syndrome” including other exploratory or risk-taking behaviors. However, this may be an advantage for the study of novel object recognition memory in fish. Because this task is typically used to measure memory rather than general activity, reactivity, or anxiety, it is ideal if object exploration does not correlate strongly with these other constructs.

Experiments on invertebrate exploration of objects are quite few. Ants, cockroaches and crickets all explore novel objects in their environment, and there is some indication that the degree of exploration is correlated with other behaviors including aggression and antipredatory behavior (Durier and Rivault, 2002; Wilson et al., 2010; Modlmeier and Foitzik, 2011; Modlmeier et al., 2012). Cephalopods, including octopus and cuttlefish, also explore objects and might be especially interesting subjects due to their sophisticated visual and tactile systems (Mather and Anderson, 1999). In general, however, the exploration of novel objects by invertebrates is still quite unexplored and a potentially interesting subject of future research.

#### Object Attributes

Exploration of objects is also affected by the properties of the object itself. Object size, complexity, stimulus qualities (e.g., texture, odor, material, movement), and affordances may all influence the latency and duration of exploration. So far, most studies on object exploration in non-mammalian species appear to have selected objects either at random, or based on unpublished past experience indicating to which objects the subject is likely to respond. No systematic analysis of how stimulus features affect exploration is available in non-mammalian species, and only limited data are available with mammals (e.g., Kornum et al., 2007; Heyser and Chemero, 2012). A few studies do validate the unsurprising conclusion that for a variety of species, the specific object does matter (Toms and Echevarria, 2014). For example, complex objects are explored more by birds than simple objects (Biondi et al., 2015), and steep objects are explored more by drosophila than shallower objects (Robie et al., 2010).

#### Context

Finally, the context in which objects are encountered affects exploration (e.g., Barnett, 1958, 1975). In rodents, the novel object test is typically conducted in an experimental arena; while most rats and mice readily explore a novel object in an unfamiliar location, objects in the home environment may be avoided and buried (Misslin and Ropartz, 1981). In non-traditional models, object exploration has been studied almost equally in home environments and novel test environments. Often, ecologists interested in neophobia place the novel object near a familiar feeder (e.g., Mettke-Hofmann et al., 2005; Funghi et al., 2015), while those interested in exploratory behavior place the object in a neutral or novel experimental arena (Stöwe et al., 2006; Lucon-Xiccato and Dadda, 2014; Toms and Echevarria, 2014). Although either context can be used for testing, the familiarity of the context clearly affects exploratory behavior in rodents (Barnett, 1958; Besheer and Bevins, 2000), and the effect of object location should be examined systematically in a new species before a standard procedure is established.

Social context may also affect exploratory behavior. Ravens approach novel objects significantly faster when tested alone than in a social dyad. However, in a social context ravens spent more time manipulating the novel object. Additionally, while there were no basic sex differences in exploration, these emerged in a social setting; males approached novel objects significantly faster than females in a social dyad (Stöwe et al., 2006). In highly social fish species such as zebrafish, it is likely that social context would matter as well, but this has not yet been tested.

### Object Learning

Outside of the pigeon literature in which explicit reinforcement is used, very few descriptions are available of the familiarization or learning process. Although the time course of familiarization was not measured with ravens, a generalization of habituation effect was observed in that across repeated tests with different objects, ravens spent significantly more time exploring the first object than subsequent objects (Stöwe et al., 2006). In raptors, habituation is apparent to simple objects with extended exploration of more complex objects. For example, Biondi et al. (2015) found that raptors who are previously exposed to simple and complex objects spend less time than controls exploring simple objects in a test, but explore complex objects almost as long as controls.

Miletto Petrazzini et al. (2012) found that newborn (4 day old) guppies significantly avoid an object when placed into a tank to which they have been familiarized for 20 h. This avoidance lasted for only about the first 5 min of a 20-min exposure trial, although a detailed analysis of time was not presented (Miletto Petrazzini et al., 2012). In contrast, Lucon-Xiccato and Dadda (2014) observed a nonsignificant tendency for adult zebrafish to approach the location of a novel object in the first 5 min of a 25-min trial. Although there was no significant approach to the object, trend analysis did reveal a significant reduction in proximity to the stimulus over time, consistent with some form of familiarization to the object (Lucon-Xiccato and Dadda, 2014). With larval zebrafish (10 days post fertilization), Andersson et al. (2015) found a significant initial bias to view a novel object with the left eye, which shifted to a right eye bias over the course of the 8-min exposure session. When tested with the same object one or two hours later, no left eye bias was observed, but when tested 3 h later a left eye bias was once again present during the first few minutes of the session. The researchers hypothesize that a left eye viewing bias is associated with novelty, and that a shift to right eye viewing may indicate familiarization with the object (Andersson et al., 2015).

### Recognition Memory

Like familiarization, recognition memory of objects has been studied to a very limited extent outside of the context of operant conditioning. Miletto Petrazzini et al. (2012) found that 4-day-old guppies spent significantly less time near a novel object than a familiar object in a preference test 30 min after exposure. Lucon-Xiccato found that in a recognition test either 2, 6, or 24 h after exposure, adult zebrafish showed a non-significant preference for a novel object over a familiar object, and a significant reduction in proximity to the novel object over the first 5 min of testing (Lucon-Xiccato and Dadda, 2014). Braida demonstrated that zebrafish spent significantly more time in proximity to a novel than a familiar shape on a video display when tested 5 min and 3 h after initial exposure, with a decrement in discrimination at 24 h and no evidence of discrimination at 96 h (Braida et al., 2014). Despite the mixed results, a consistent theme does emerge; the animal’s response to the novel object in a test resembles its initial behavior toward the sample object in the exposure phase, suggesting that further study on the mechanisms of habituation and perception in these animals will be useful for studying recognition memory as well.

## Design Considerations

The novel object recognition test is advantageous due in part to its procedural simplicity, but this simplicity unfortunately does not extend to the experimental design. Several relevant factors must be considered and most likely examined in pilot studies prior to validating this procedure for a new species. These considerations include: object selection, behavioral measures, trial duration, control stimuli or groups, and statistical analysis.

### Object Selection

Even in the extensive rodent literature, it is not clear what distinguishes an “object” from other features of the environment like an arena wall (Gibson, 1979). In practice, an object is typically a three-dimensional mass that can be directly approached and perceived through more than one sensory modality (e.g., visual and tactile). The size of the object relative to the body of the animal almost certainly affects exploratory behavior, as well as whether the animal can move, climb on, hide under, or manipulate the object in some other way (i.e., object affordances: Gibson, 1979; Ennaceur, 2010; Heyser and Chemero, 2012). Objects may have multimodal features, including odors, textures, and even movement, which can substantially affect exploration and should therefore be selected in light of the subject’s sensory system and the experimental question. Unlearned object preferences are commonly observed across a variety of species; for example, zebrafish may prefer stimuli that reflect slow-wavelength light (blue and purple; Colwill et al., 2005), octopusus prefer dark to light shapes (Messenger and Sanders, 1972), and jackdaws and ravens prefer to manipulate and cache spherically-shaped objects (Jacobs et al., 2014). Because of such preferences, detailed measurement and reporting of object characteristics is essential (as an example, see; Kornum et al., 2007 with Gottinger minipigs). Even after preliminary testing, objects should always be counterbalanced such that each object is equally often used as the novel and the familiar object. If objects are asymmetrical, the orientation of the object relative to the arena must also be consistent, lest an unfamiliar perspective on the object relative to background be perceived as a novel object. Because location biases may also exist, the location of the novel object should also be balanced. Potential marking stimuli (e.g., odors) must also be eliminated between trials, both on the objects and in the testing arena.

### Behavioral Measures

In birds, exploration is most often measured as approach latency, frequently including duration in proximity to an object, and occasionally also duration in contact with the object. In fish, object exploration is often measured simply on the basis of proximity to the object, sometimes with head orientation included (Burns, 2008; Braida et al., 2014). However, given the lateral orientation of zebrafish eyes and the possibility of lateralization of function, it is unclear what head orientation should be taken to indicate “exploration” of an object (Andersson et al., 2015). Rats, who have rather poor vision, tend to approach and manipulate novel objects, receiving olfactory and tactile information through the nose, paws, and vibrissae, and exploration is usually defined on the basis of nose proximity (Ennaceur, 2010). Exploratory birds also tend to approach novel objects and manipulate them with their bill and feet, which receive tactile information (Demery et al., 2011). Fish, in contrast, live in an aquatic environment and do not have sensory organs that are equivalent to vibrissa, bill tips, or feet. Tactile information can potentially be received by the skin or lateral line organs at some distance from the object, through water movement. It might therefore be possible for zebrafish to identify an object using visual, tactile, and/or chemosensory cues without coming into close proximity to that object. Similarly, animals that gain information through electrolocation, echolocation, or comparable mechanisms may not need to approach objects closely for recognition to occur. Therefore, validation of any selected measure of exploration is imperative. Ideally, a relationship should be detectable between the amount of time spent “exploring” an object using a given behavioral measure, and subsequent memory for the object. Less strictly, some evidence that the exploration measure affects familiarization and memory is requisite—for example, discrimination should fall to chance if the exploratory behavior is prevented entirely. Automated behavioral measurement can be used to record some behaviors, such as latency to approach and proximity to the object, and it has the dual advantages of being fully objective and highly efficient. However, there are still some behavioral measures that cannot be detected by current automated systems, especially in non-traditional species. Therefore, manual behavioral recording may be necessary and should always be done by experimentally blind observers in order to reduce experimenter bias and provide evidence of reliability.

### Trial and Inter-Trial Duration

The duration of the sample exposure can be a single continuous trial or divided into multiple trials. One issue with using a set trial duration (e.g., 6 min) is that animals will inherently differ in the amount of sample exploration and this may affect the strength of recognition memory. This is especially problematic if the experimenter wishes to compare memory across two groups that may differ in baseline exploration (e.g., migratory and resident species of birds). An alternative method is to allow the organism as much time as required to reach a sample object exploration criterion (e.g., 30 s) (e.g., Norman and Eacott, 2004). Whereas this does equate sample exploration across subjects, it can confound exposure to the context and significantly lengthen the duration of the experiment. If multiple trials are to be used, an inter-trial interval needs to be selected. Typically in rodents, the ITI is short (minutes), but this will require validation across individual species. These procedural variables significantly affect object recognition performance, as highlighted in a recent report showing that spaced training rescues memory and extracellular-signal-regulated kinases (ERK1/2) signaling in fragile X syndrome model mice (Seese et al., 2014). In that study, wild-type mice exhibited robust object location memory using a single sample trial, whereas fragile X mental retardation 1 knockout (Fmr1 KO) mice did not. However, object location memory was observed in Fmr1 KO mice if the mice received the same amount of training distributed across three short trials (Seese et al., 2014).

### Controls

Most often, the novel object recognition test is designed as a within-subjects test, with the experimental (novel) stimulus and the control (familiar) stimulus presented simultaneously to each subject. This is useful when examining additional between-subjects variables such as the effects of lesions, genetic manipulations, or pharmacological treatments on recognition memory. However, the test can also use a between-subjects design, with two groups receiving an exposure trial with an object, followed by testing in the experimental group with a novel object and the control group with the familiar object. With proper selection and counterbalancing of stimuli, and elimination of any potential marking cues, any significant difference in responding to the novel and familiar objects indicates some form of memory. However, if a lesion (for example) disrupts discrimination between objects, this could be due to disrupted memory, or alternatively to a disruption in sensory/perceptual abilities, locomotor activity, fear/anxiety, or novelty-directed motivation. A reduction in discrimination can also result from dishabituation to the familiar stimulus resulting from exposure to the novel stimulus (or depending on the experiment, to a novel context or spatial arrangement). Supporting evidence that these alternative processes remain normal in experimental animals is necessary to confidently attribute alterations in this task to memory effects.

### Statistical Analysis

Because the recognition test is usually a two-choice test, a measure of discrimination is typically used for analysis. Several discrimination measures have been used in the rodent literature, including a simple difference score (novel exploration − familiar exploration), a relative difference score [(novel exploration − familiar exploration)/familiar exploration), or a ratio score (novel exploration/(novel exploration + familiar exploration)]. The latter two options help to correct for differences in total exploration, and therefore yield different outcomes from the former (Akkerman et al., 2012). Although it is not in widespread practice, discrimination performance should be statistically compared to chance (no discrimination) in order to establish that significant discrimination between the objects did occur (Akkerman et al., 2012).

## Conclusion

Object exploration and recognition provide a potentially useful direction for the comparative study of recognition memory. Research on novel object exploration in non-mammalian models has so far been basically limited to examination of neophobia from an ecological perspective in birds, and individual differences in fish behavior. Therefore, while a strong foundation is in place from research on visual perception, neophobia, and exploratory behaviors in many species, the question of recognition memory is still largely unexplored. Because of this, considerable work is still needed to develop valid, reliable, and species-appropriate procedures. Systematic examination is required of exploratory behaviors in home and novel contexts, the role of object features such as size, motion, and location, and in some cases the perceptual abilities of the animal. The necessary parametric work may seem daunting, but given the popularity of this test in rodents, the rewards could be substantial.

The goal of this review has been to highlight the advantages and methodological considerations of the spontaneous object recognition test for researchers using non-mammalian species. It is common practice in the neurosciences to compare distantly related species sharing a trait of interest, with the expectation that the neurological mechanisms of that trait have some degree of homology. One example of this approach is the study of cholinergic involvement in learning and memory (Easton and Eacott, 2013). In humans, dysfunction of the acetylcholine system is involved in the cognitive decline observed in Schizophrenia, Alzheimer’s, and other forms of dementia (Lyon et al., 2012). Most often, rodent models have been used to study the neurobiology of these disorders. However, the cholinergic system is also involved in learning in zebrafish (Bortolotto et al., 2015) and a variety of invertebrates, including mollusks (Mpitsos et al., 1988; Fiorito et al., 1998), crustaceans (Caffaro et al., 2012) and insects (Barraco and Eisenstein, 1984; Terazima and Yoshino, 2010). Given the proven utility of non-mammalian species for genetic, developmental, pharmacological, and synaptic-level analysis, we contend that the inclusion of these species will enhance our understanding of the neurobiology of recognition memory (Dere et al., 2007; Warburton and Brown, 2015). To this end, the spontaneous object recognition test is likely to be a versatile tool for translational research with non-mammalian models.

## Conflict of Interest Statement

The authors declare that the research was conducted in the absence of any commercial or financial relationships that could be construed as a potential conflict of interest.
